# *trans*-Bis{1,2-bis­[bis­(2-methoxy­ethyl)phosphino]ethane}dichloridoiron(II)

**DOI:** 10.1107/S1600536810003727

**Published:** 2010-02-06

**Authors:** Justin L. Crossland, Lev N. Zakharov, David R. Tyler

**Affiliations:** a Department of Chemistry, 1253 University of Oregon, Eugene, Oregon 97403-1253, USA

## Abstract

The Fe atom in the title compound, [FeCl_2_(C_14_H_32_O_4_P_2_)_2_], has a distorted octa­hedral coordination with four P atoms in equatorial positions and two Cl atoms in apical positions.

## Related literature

For the applications of similar complexes in dinitro­gen binding, see: Gilbertson *et al.* (2007 ▶); Lyon (1993 ▶); MacKay & Fryzuk (2004 ▶). For related structures, see: Herbowski & Deutsch (1993 ▶); Miller *et al.* (2002 ▶).
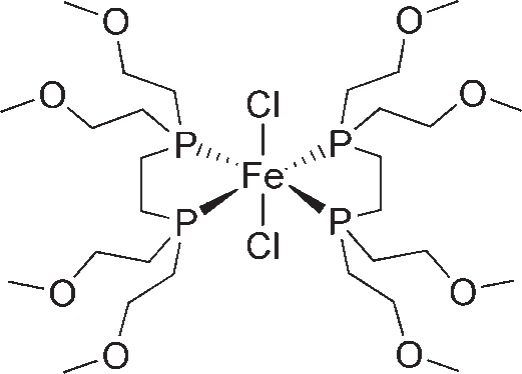


## Experimental

### 

#### Crystal data

[FeCl_2_(C_14_H_32_O_4_P_2_)_2_]
*M* *_r_* = 779.42
Monoclinic, 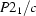

*a* = 12.3417 (7) Å
*b* = 12.1825 (7) Å
*c* = 25.3621 (15) Å
β = 100.124 (1)°
*V* = 3753.9 (4) Å^3^
*Z* = 4
Mo *K*α radiation
μ = 0.76 mm^−1^
*T* = 173 K
0.32 × 0.19 × 0.09 mm

#### Data collection

Bruker APEX CCD area-detector diffractometer
Absorption correction: multi-scan (*SADABS*; Sheldrick, 1995 ▶) *T*_min_ = 0.794, *T*_max_ = 0.935
41489 measured reflections
8193 independent reflections
7057 reflections with *I* > 2σ(*I*)
*R*_int_ = 0.033

#### Refinement

*R*[*F* ^2^ > 2σ(*F* ^2^)] = 0.034
*wR*(*F* ^2^) = 0.094
*S* = 1.03
8193 reflections
388 parameters
H-atom parameters constrained
Δρ_max_ = 1.70 e Å^−3^
Δρ_min_ = −0.52 e Å^−3^

### 

Data collection: *SMART* (Bruker, 2000 ▶); cell refinement: *SAINT* (Bruker, 2000 ▶); data reduction: *SAINT*; program(s) used to solve structure: *SHELXTL* (Sheldrick, 2008 ▶); program(s) used to refine structure: *SHELXTL*; molecular graphics: *SHELXTL*; software used to prepare material for publication: *SHELXTL*.

## Supplementary Material

Crystal structure: contains datablocks I, global. DOI: 10.1107/S1600536810003727/sj2718sup1.cif

Structure factors: contains datablocks I. DOI: 10.1107/S1600536810003727/sj2718Isup2.hkl

Additional supplementary materials:  crystallographic information; 3D view; checkCIF report

## Figures and Tables

**Table:** 

Fe1—P1	2.2581 (6)
Fe1—P2	2.2770 (5)
Fe1—P3	2.2792 (6)
Fe1—P4	2.2814 (5)
Fe1—Cl2	2.3491 (5)
Fe1—Cl1	2.3529 (5)

**Table:** 

P1—Fe1—P3	175.71 (2)
P2—Fe1—P4	178.82 (2)
Cl2—Fe1—Cl1	179.11 (2)

**Fig. 1.**
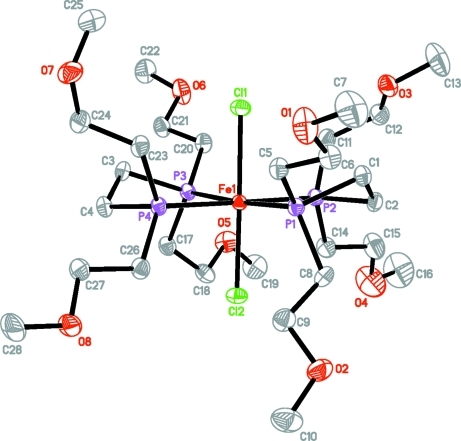
The structure of the title compound with 50% probability displacement ellipsoids and the atom-numbering scheme. The H atoms are omitted for clarity.

## supplementary crystallographic information

### Comment

Numerous iron-diphosphine complexes have shown the ability to coordinate dinitrogen (MacKay & Fryzuk, 2004). Because of this ability, these complexes have received interest as dinitrogen scrubbers for nitrogen-containing natural gas streams (Lyon, 1993). One requirement for a successful dinitrogen scrubber is high solubility in water, a solvent in which methane has limited solubility. Research in our group has explored the synthesis of iron-diphosphine complexes containing water-soluble phosphine ligands, specifically diphosphine ligands containing hydroxyl and methoxy functional groups (Gilbertson *et al.*, 2007; Miller *et al.*, 2002). One problem facing hydroxyl functionalized phosphine ligands is that the hydroxyl groups have been shown in some cases to coordinate to the metal center. The methoxy functionalized phosphines are not plagued by this problem and thus have been our recent focus. Here we report the synthesis and structural characterization of a water-soluble iron dichloride phosphine complex, *trans*-Fe(DMeOEtPE)_2_Cl_2_ (DMeOEtPE=1,2-bis(dimethoxyethylphosphino)ethane).

The Fe atom in *trans*-Fe(DMeOEtPE)_2_Cl_2_ has a distorted octahedral coordination with four P atoms in equatorial and two Cl atoms in apical positions (Fig. 1). The Fe(1)—P distances are in the range 2.2581 (6)-2.2814 (5) Å, and the Fe(1)—Cl(1,2) distances are 2.3529 (5) and 2.3491 (5) Å, respectively.

### Experimental

1,2-bis(dimethoxyethylphosphino)ethane (DMeOEtPE) was synthesized as previously reported (Herbowski & Deutsch, 1993). *trans*-Fe(DMeOEtPE)_2_Cl_2_ was prepared by adding DMeOEtPE (0.826 g, 2.53 mmol) to a stirring solution of FeCl_2_4H_2_O (0.25 g, 1.26 mmol) in 30 ml of toluene under an argon atmosphere. The reaction was allowed to stir at room temperature for 24 hrs. The resulting green solution was carefully decanted into a clean flask, leaving a small amount of oily, red impurity in the original vessel. Approximately 20 ml of the toluene was removed under vacuum followed by addition of hexane (50 ml). Vacuum was applied to remove some of the hexane and chill the mixture. A green crystalline product was obtained by filtration followed by a hexane rinse and drying in vacuo. Yield 0.73 g, 74%. ^31^P{^1^H} NMR (toluene-d^8^) at 233 K: 55 ppm.

### Refinement

The H atoms were positioned geometrically and refined using the riding model approximation, C—H = 0.99 and 0.98 Å; U_iso_(H) = 1.2U_eq_(C) and 1.5U_eq_(C), respectively for CH_2_ and CH_3_ groups. There are eight flexible (CH_2_CH_2_OCH_3_) groups in the structure and as a result there are elongations of displacement ellipsoids for some atoms. On the residial density there is one peak, 1.699 e Å^3^, corresponding to a second position for the O(4) atom. The treatment of the disorder shows that the O(4) atom in the terminal C(15)O(4) C(16) group is disordered over two postions in ratio 84/16. Such refinement doesn't significantly improve the final crystal structure and the second possible position for the disordered C(15)O(4) C(16) group was not taken into consideration.

### Crystal data

**Table:**

[FeCl_2_(C_14_H_32_O_4_P_2_)_2_]	*F*(000) = 1664
*M**_r_* = 779.42	*D*_x_ = 1.379 Mg m^−^^3^
Monoclinic, *P*2_1_/*c*	Mo *K*α radiation, λ = 0.71073 Å
Hall symbol: -P 2ybc	Cell parameters from 5483 reflections
*a* = 12.3417 (7) Å	θ = 2.3–27.0°
*b* = 12.1825 (7) Å	µ = 0.76 mm^−^^1^
*c* = 25.3621 (15) Å	*T* = 173 K
β = 100.124 (1)°	Block, light green
*V* = 3753.9 (4) Å^3^	0.32 × 0.19 × 0.09 mm
*Z* = 4	

### Data collection

**Table:**

Bruker APEX CCD area-detector diffractometer	8193 independent reflections
Radiation source: fine-focus sealed tube	7057 reflections with *I* > 2σ(*I*)
graphite	*R*_int_ = 0.033
φ and ω scans	θ_max_ = 27.0°, θ_min_ = 1.6°
Absorption correction: multi-scan (*SADABS*; Sheldrick, 1995)	*h* = −15→15
*T*_min_ = 0.794, *T*_max_ = 0.935	*k* = −15→15
41489 measured reflections	*l* = −32→32

### Refinement

**Table:**

Refinement on *F*^2^	Primary atom site location: structure-invariant direct methods
Least-squares matrix: full	Secondary atom site location: difference Fourier map
*R*[*F*^2^ > 2σ(*F*^2^)] = 0.034	Hydrogen site location: inferred from neighbouring sites
*wR*(*F*^2^) = 0.094	H-atom parameters constrained
*S* = 1.03	*w* = 1/[σ^2^(*F*_o_^2^) + (0.0481*P*)^2^ + 2.6254*P*] where *P* = (*F*_o_^2^ + 2*F*_c_^2^)/3
8193 reflections	(Δ/σ)_max_ = 0.001
388 parameters	Δρ_max_ = 1.70 e Å^−^^3^
0 restraints	Δρ_min_ = −0.52 e Å^−^^3^

### Special details

**Table:**

Geometry. All esds (except the esd in the dihedral angle between two l.s. planes) are estimated using the full covariance matrix. The cell esds are taken into account individually in the estimation of esds in distances, angles and torsion angles; correlations between esds in cell parameters are only used when they are defined by crystal symmetry. An approximate (isotropic) treatment of cell esds is used for estimating esds involving l.s. planes.
Refinement. Refinement of *F*^2^ against ALL reflections. The weighted *R*-factor wR and goodness of fit *S* are based on *F*^2^, conventional *R*-factors *R* are based on *F*, with *F* set to zero for negative *F*^2^. The threshold expression of *F*^2^ > σ(*F*^2^) is used only for calculating *R*-factors(gt) etc. and is not relevant to the choice of reflections for refinement. *R*-factors based on *F*^2^ are statistically about twice as large as those based on *F*, and *R*- factors based on ALL data will be even larger.

### Fractional atomic coordinates and isotropic or equivalent isotropic displacement parameters (Å^2^)

**Table:**

	*x*	*y*	*z*	*U*_iso_*/*U*_eq_	
Fe1	0.22245 (2)	0.97476 (2)	0.194352 (10)	0.01452 (8)	
Cl1	0.08642 (4)	0.83773 (4)	0.187910 (19)	0.02216 (11)	
Cl2	0.35615 (4)	1.11358 (4)	0.200262 (19)	0.02214 (11)	
P1	0.30134 (4)	0.87521 (4)	0.136197 (19)	0.01708 (11)	
P2	0.33088 (4)	0.87234 (4)	0.257867 (19)	0.01768 (11)	
P3	0.13372 (4)	1.06472 (4)	0.253109 (19)	0.01742 (11)	
P4	0.11197 (4)	1.07416 (4)	0.130031 (19)	0.01745 (11)	
O1	0.19237 (15)	0.71639 (14)	−0.00460 (6)	0.0400 (4)	
O2	0.54120 (13)	1.03024 (14)	0.07202 (7)	0.0341 (4)	
O3	0.26896 (14)	0.60274 (13)	0.26502 (7)	0.0352 (4)	
O4	0.57019 (19)	0.95126 (18)	0.38323 (9)	0.0639 (6)	
O5	0.27939 (14)	1.12839 (14)	0.37116 (6)	0.0341 (4)	
O6	−0.03861 (15)	0.96739 (14)	0.36821 (7)	0.0377 (4)	
O7	−0.18270 (12)	0.98052 (14)	0.03537 (6)	0.0315 (4)	
O8	0.15366 (13)	1.29630 (13)	0.01486 (6)	0.0295 (3)	
C1	0.35201 (17)	0.74704 (16)	0.17059 (8)	0.0217 (4)	
H1A	0.2895	0.6989	0.1748	0.026*	
H1B	0.4001	0.7071	0.1497	0.026*	
C2	0.41655 (16)	0.77930 (17)	0.22520 (8)	0.0213 (4)	
H2A	0.4858	0.8165	0.2209	0.026*	
H2B	0.4355	0.7130	0.2475	0.026*	
C3	0.00598 (16)	1.12040 (17)	0.21359 (8)	0.0217 (4)	
H3A	−0.0480	1.0606	0.2034	0.026*	
H3B	−0.0269	1.1751	0.2350	0.026*	
C4	0.03478 (17)	1.17450 (17)	0.16345 (8)	0.0222 (4)	
H4A	0.0801	1.2408	0.1734	0.027*	
H4B	−0.0333	1.1967	0.1390	0.027*	
C5	0.21964 (16)	0.82790 (17)	0.07278 (8)	0.0214 (4)	
H5A	0.1470	0.8038	0.0800	0.026*	
H5B	0.2066	0.8916	0.0483	0.026*	
C6	0.26683 (17)	0.73570 (17)	0.04360 (8)	0.0245 (4)	
H6A	0.3398	0.7568	0.0358	0.029*	
H6B	0.2756	0.6685	0.0659	0.029*	
C7	0.2245 (3)	0.6320 (2)	−0.03610 (11)	0.0463 (7)	
H7A	0.1692	0.6236	−0.0687	0.069*	
H7B	0.2310	0.5632	−0.0158	0.069*	
H7C	0.2957	0.6503	−0.0459	0.069*	
C8	0.43141 (16)	0.92093 (18)	0.11705 (8)	0.0241 (4)	
H8A	0.4839	0.9362	0.1505	0.029*	
H8B	0.4615	0.8584	0.0993	0.029*	
C9	0.43092 (18)	1.01955 (19)	0.08110 (9)	0.0274 (5)	
H9A	0.3796	1.0081	0.0469	0.033*	
H9B	0.4081	1.0862	0.0987	0.033*	
C10	0.5572 (2)	1.1256 (2)	0.04272 (10)	0.0396 (6)	
H10A	0.6340	1.1288	0.0375	0.059*	
H10B	0.5405	1.1906	0.0625	0.059*	
H10C	0.5084	1.1234	0.0078	0.059*	
C11	0.26345 (18)	0.78176 (18)	0.30135 (9)	0.0267 (5)	
H11A	0.2683	0.8187	0.3365	0.032*	
H11B	0.1844	0.7774	0.2854	0.032*	
C12	0.30510 (19)	0.66529 (18)	0.31191 (9)	0.0305 (5)	
H12A	0.3865	0.6648	0.3207	0.037*	
H12B	0.2758	0.6336	0.3425	0.037*	
C13	0.3024 (3)	0.4922 (2)	0.27114 (13)	0.0592 (9)	
H13A	0.2757	0.4521	0.2379	0.089*	
H13B	0.2719	0.4590	0.3005	0.089*	
H13C	0.3830	0.4885	0.2793	0.089*	
C14	0.43117 (18)	0.94582 (18)	0.30686 (9)	0.0277 (5)	
H14A	0.4756	0.9927	0.2869	0.033*	
H14B	0.3900	0.9954	0.3272	0.033*	
C15	0.5095 (2)	0.8796 (2)	0.34673 (10)	0.0425 (6)	
H15A	0.4679	0.8278	0.3657	0.051*	
H15B	0.5594	0.8367	0.3281	0.051*	
C16	0.6081 (3)	0.9017 (3)	0.43294 (12)	0.0707 (10)	
H16A	0.6508	0.9551	0.4570	0.106*	
H16B	0.6549	0.8388	0.4280	0.106*	
H16C	0.5452	0.8767	0.4485	0.106*	
C17	0.18716 (18)	1.19478 (17)	0.28572 (8)	0.0245 (4)	
H17A	0.1977	1.2466	0.2570	0.029*	
H17B	0.1287	1.2259	0.3034	0.029*	
C18	0.29249 (18)	1.19403 (18)	0.32654 (8)	0.0263 (4)	
H18A	0.3533	1.1646	0.3100	0.032*	
H18B	0.3117	1.2700	0.3386	0.032*	
C19	0.3712 (2)	1.1381 (2)	0.41344 (9)	0.0408 (6)	
H19A	0.3596	1.0917	0.4435	0.061*	
H19B	0.3792	1.2147	0.4252	0.061*	
H19C	0.4380	1.1145	0.4007	0.061*	
C20	0.08209 (18)	0.98752 (17)	0.30572 (8)	0.0233 (4)	
H20A	0.0556	0.9153	0.2908	0.028*	
H20B	0.1447	0.9737	0.3352	0.028*	
C21	−0.00960 (18)	1.04069 (18)	0.32942 (9)	0.0263 (5)	
H21A	0.0156	1.1115	0.3464	0.032*	
H21B	−0.0740	1.0548	0.3010	0.032*	
C22	−0.1338 (2)	1.0037 (2)	0.38722 (10)	0.0391 (6)	
H22A	−0.1518	0.9515	0.4138	0.059*	
H22B	−0.1956	1.0084	0.3572	0.059*	
H22C	−0.1199	1.0761	0.4038	0.059*	
C23	0.00492 (16)	0.99868 (17)	0.08353 (8)	0.0228 (4)	
H23A	0.0312	0.9902	0.0491	0.027*	
H23B	−0.0010	0.9241	0.0983	0.027*	
C24	−0.11012 (17)	1.04770 (19)	0.07170 (9)	0.0262 (4)	
H24A	−0.1391	1.0552	0.1055	0.031*	
H24B	−0.1066	1.1218	0.0561	0.031*	
C25	−0.2284 (2)	0.8934 (2)	0.06156 (10)	0.0376 (6)	
H25A	−0.2776	0.8497	0.0350	0.056*	
H25B	−0.2702	0.9237	0.0877	0.056*	
H25C	−0.1691	0.8466	0.0800	0.056*	
C26	0.17307 (17)	1.16139 (17)	0.08401 (8)	0.0228 (4)	
H26A	0.2262	1.2118	0.1055	0.027*	
H26B	0.2150	1.1140	0.0631	0.027*	
C27	0.09244 (18)	1.22924 (18)	0.04524 (9)	0.0262 (5)	
H27A	0.0422	1.1805	0.0211	0.031*	
H27B	0.0477	1.2758	0.0652	0.031*	
C28	0.0831 (2)	1.3495 (2)	−0.02759 (10)	0.0394 (6)	
H28A	0.1271	1.3948	−0.0478	0.059*	
H28B	0.0310	1.3961	−0.0129	0.059*	
H28C	0.0426	1.2943	−0.0514	0.059*	

### Atomic displacement parameters (Å^2^)

**Table:**

	*U*^11^	*U*^22^	*U*^33^	*U*^12^	*U*^13^	*U*^23^
Fe1	0.01472 (14)	0.01411 (14)	0.01440 (14)	−0.00007 (10)	0.00165 (10)	0.00078 (10)
Cl1	0.0221 (2)	0.0208 (2)	0.0230 (2)	−0.00560 (18)	0.00258 (18)	0.00148 (18)
Cl2	0.0229 (2)	0.0229 (2)	0.0202 (2)	−0.00762 (18)	0.00275 (18)	−0.00048 (18)
P1	0.0166 (2)	0.0176 (2)	0.0168 (2)	0.00109 (18)	0.00235 (18)	−0.00022 (18)
P2	0.0182 (2)	0.0183 (2)	0.0160 (2)	0.00186 (19)	0.00133 (19)	0.00167 (18)
P3	0.0191 (2)	0.0169 (2)	0.0163 (2)	0.00138 (19)	0.00322 (19)	0.00023 (18)
P4	0.0183 (2)	0.0175 (2)	0.0160 (2)	0.00196 (19)	0.00166 (18)	0.00139 (18)
O1	0.0503 (11)	0.0377 (10)	0.0264 (8)	0.0136 (8)	−0.0088 (8)	−0.0137 (7)
O2	0.0260 (8)	0.0392 (9)	0.0398 (9)	−0.0014 (7)	0.0135 (7)	0.0062 (7)
O3	0.0346 (9)	0.0241 (8)	0.0437 (10)	0.0014 (7)	−0.0018 (7)	0.0085 (7)
O4	0.0736 (15)	0.0545 (13)	0.0500 (13)	−0.0061 (11)	−0.0268 (11)	−0.0005 (10)
O5	0.0364 (9)	0.0427 (10)	0.0219 (8)	−0.0054 (7)	0.0012 (7)	0.0022 (7)
O6	0.0466 (10)	0.0339 (9)	0.0398 (10)	0.0134 (8)	0.0277 (8)	0.0145 (7)
O7	0.0244 (8)	0.0394 (9)	0.0278 (8)	−0.0064 (7)	−0.0036 (6)	0.0024 (7)
O8	0.0307 (8)	0.0320 (8)	0.0246 (8)	−0.0033 (7)	0.0017 (6)	0.0117 (6)
C1	0.0250 (10)	0.0188 (10)	0.0207 (10)	0.0052 (8)	0.0028 (8)	0.0005 (8)
C2	0.0187 (10)	0.0227 (10)	0.0220 (10)	0.0053 (8)	0.0025 (8)	0.0028 (8)
C3	0.0197 (10)	0.0243 (10)	0.0211 (10)	0.0065 (8)	0.0039 (8)	0.0004 (8)
C4	0.0236 (10)	0.0206 (10)	0.0214 (10)	0.0055 (8)	0.0014 (8)	0.0012 (8)
C5	0.0214 (10)	0.0231 (10)	0.0189 (10)	0.0025 (8)	0.0011 (8)	−0.0026 (8)
C6	0.0275 (11)	0.0246 (11)	0.0212 (10)	0.0006 (9)	0.0037 (8)	−0.0025 (8)
C7	0.0630 (18)	0.0407 (15)	0.0338 (14)	0.0007 (13)	0.0046 (13)	−0.0168 (11)
C8	0.0185 (10)	0.0291 (11)	0.0253 (11)	0.0011 (8)	0.0056 (8)	−0.0006 (9)
C9	0.0239 (11)	0.0335 (12)	0.0262 (11)	0.0010 (9)	0.0084 (9)	0.0023 (9)
C10	0.0457 (15)	0.0380 (14)	0.0393 (14)	−0.0083 (11)	0.0187 (12)	0.0014 (11)
C11	0.0253 (11)	0.0299 (12)	0.0258 (11)	0.0038 (9)	0.0074 (9)	0.0096 (9)
C12	0.0298 (12)	0.0294 (12)	0.0323 (12)	0.0027 (9)	0.0057 (9)	0.0131 (9)
C13	0.081 (2)	0.0315 (15)	0.062 (2)	0.0152 (15)	0.0033 (17)	0.0078 (14)
C14	0.0305 (12)	0.0256 (11)	0.0232 (11)	0.0013 (9)	−0.0056 (9)	−0.0031 (9)
C15	0.0469 (15)	0.0352 (14)	0.0361 (14)	0.0069 (11)	−0.0184 (11)	−0.0024 (11)
C16	0.084 (3)	0.079 (2)	0.0369 (16)	0.004 (2)	−0.0216 (16)	0.0008 (16)
C17	0.0288 (11)	0.0191 (10)	0.0256 (11)	0.0014 (8)	0.0049 (9)	−0.0036 (8)
C18	0.0327 (12)	0.0220 (10)	0.0239 (11)	−0.0015 (9)	0.0036 (9)	−0.0031 (8)
C19	0.0482 (15)	0.0464 (15)	0.0239 (12)	−0.0013 (12)	−0.0041 (11)	−0.0022 (11)
C20	0.0289 (11)	0.0212 (10)	0.0207 (10)	0.0035 (8)	0.0075 (8)	0.0027 (8)
C21	0.0296 (11)	0.0262 (11)	0.0251 (11)	0.0060 (9)	0.0102 (9)	0.0056 (9)
C22	0.0386 (14)	0.0470 (15)	0.0361 (13)	0.0066 (12)	0.0183 (11)	0.0061 (11)
C23	0.0222 (10)	0.0234 (10)	0.0206 (10)	0.0021 (8)	−0.0026 (8)	−0.0019 (8)
C24	0.0214 (10)	0.0299 (11)	0.0256 (11)	0.0013 (9)	−0.0008 (8)	0.0009 (9)
C25	0.0300 (13)	0.0387 (14)	0.0448 (15)	−0.0063 (10)	0.0083 (11)	−0.0005 (11)
C26	0.0230 (10)	0.0253 (10)	0.0201 (10)	0.0031 (8)	0.0033 (8)	0.0070 (8)
C27	0.0263 (11)	0.0274 (11)	0.0245 (11)	0.0011 (9)	0.0030 (8)	0.0080 (9)
C28	0.0464 (15)	0.0368 (14)	0.0329 (13)	0.0002 (11)	0.0009 (11)	0.0169 (11)

### Geometric parameters (Å, °)

**Table:**

Fe1—P1	2.2581 (6)	C8—H8B	0.9900
Fe1—P2	2.2770 (5)	C9—H9A	0.9900
Fe1—P3	2.2792 (6)	C9—H9B	0.9900
Fe1—P4	2.2814 (5)	C10—H10A	0.9800
Fe1—Cl2	2.3491 (5)	C10—H10B	0.9800
Fe1—Cl1	2.3529 (5)	C10—H10C	0.9800
P1—C5	1.835 (2)	C11—C12	1.517 (3)
P1—C8	1.843 (2)	C11—H11A	0.9900
P1—C1	1.843 (2)	C11—H11B	0.9900
P2—C14	1.827 (2)	C12—H12A	0.9900
P2—C2	1.843 (2)	C12—H12B	0.9900
P2—C11	1.857 (2)	C13—H13A	0.9800
P3—C20	1.836 (2)	C13—H13B	0.9800
P3—C3	1.842 (2)	C13—H13C	0.9800
P3—C17	1.854 (2)	C14—C15	1.505 (3)
P4—C26	1.836 (2)	C14—H14A	0.9900
P4—C4	1.845 (2)	C14—H14B	0.9900
P4—C23	1.853 (2)	C15—H15A	0.9900
O1—C7	1.401 (3)	C15—H15B	0.9900
O1—C6	1.414 (2)	C16—H16A	0.9800
O2—C10	1.411 (3)	C16—H16B	0.9800
O2—C9	1.426 (3)	C16—H16C	0.9800
O3—C13	1.409 (3)	C17—C18	1.513 (3)
O3—C12	1.417 (3)	C17—H17A	0.9900
O4—C15	1.391 (3)	C17—H17B	0.9900
O4—C16	1.402 (4)	C18—H18A	0.9900
O5—C18	1.418 (3)	C18—H18B	0.9900
O5—C19	1.421 (3)	C19—H19A	0.9800
O6—C22	1.417 (3)	C19—H19B	0.9800
O6—C21	1.420 (3)	C19—H19C	0.9800
O7—C25	1.421 (3)	C20—C21	1.517 (3)
O7—C24	1.425 (3)	C20—H20A	0.9900
O8—C28	1.417 (3)	C20—H20B	0.9900
O8—C27	1.427 (2)	C21—H21A	0.9900
C1—C2	1.523 (3)	C21—H21B	0.9900
C1—H1A	0.9900	C22—H22A	0.9800
C1—H1B	0.9900	C22—H22B	0.9800
C2—H2A	0.9900	C22—H22C	0.9800
C2—H2B	0.9900	C23—C24	1.521 (3)
C3—C4	1.529 (3)	C23—H23A	0.9900
C3—H3A	0.9900	C23—H23B	0.9900
C3—H3B	0.9900	C24—H24A	0.9900
C4—H4A	0.9900	C24—H24B	0.9900
C4—H4B	0.9900	C25—H25A	0.9800
C5—C6	1.517 (3)	C25—H25B	0.9800
C5—H5A	0.9900	C25—H25C	0.9800
C5—H5B	0.9900	C26—C27	1.515 (3)
C6—H6A	0.9900	C26—H26A	0.9900
C6—H6B	0.9900	C26—H26B	0.9900
C7—H7A	0.9800	C27—H27A	0.9900
C7—H7B	0.9800	C27—H27B	0.9900
C7—H7C	0.9800	C28—H28A	0.9800
C8—C9	1.508 (3)	C28—H28B	0.9800
C8—H8A	0.9900	C28—H28C	0.9800
			
P1—Fe1—P2	84.29 (2)	C12—C11—H11A	107.5
P1—Fe1—P3	175.71 (2)	P2—C11—H11A	107.5
P2—Fe1—P3	95.23 (2)	C12—C11—H11B	107.5
P1—Fe1—P4	95.17 (2)	P2—C11—H11B	107.5
P2—Fe1—P4	178.82 (2)	H11A—C11—H11B	107.0
P3—Fe1—P4	85.23 (2)	O3—C12—C11	107.94 (18)
P1—Fe1—Cl2	92.85 (2)	O3—C12—H12A	110.1
P2—Fe1—Cl2	91.75 (2)	C11—C12—H12A	110.1
P3—Fe1—Cl2	91.43 (2)	O3—C12—H12B	110.1
P4—Fe1—Cl2	89.33 (2)	C11—C12—H12B	110.1
P1—Fe1—Cl1	87.59 (2)	H12A—C12—H12B	108.4
P2—Fe1—Cl1	89.07 (2)	O3—C13—H13A	109.5
P3—Fe1—Cl1	88.14 (2)	O3—C13—H13B	109.5
P4—Fe1—Cl1	89.85 (2)	H13A—C13—H13B	109.5
Cl2—Fe1—Cl1	179.11 (2)	O3—C13—H13C	109.5
C5—P1—C8	103.52 (10)	H13A—C13—H13C	109.5
C5—P1—C1	103.72 (9)	H13B—C13—H13C	109.5
C8—P1—C1	98.16 (10)	C15—C14—P2	118.23 (16)
C5—P1—Fe1	120.51 (7)	C15—C14—H14A	107.8
C8—P1—Fe1	120.35 (7)	P2—C14—H14A	107.8
C1—P1—Fe1	107.17 (7)	C15—C14—H14B	107.8
C14—P2—C2	103.50 (10)	P2—C14—H14B	107.8
C14—P2—C11	102.01 (11)	H14A—C14—H14B	107.1
C2—P2—C11	104.70 (10)	O4—C15—C14	108.5 (2)
C14—P2—Fe1	117.16 (7)	O4—C15—H15A	110.0
C2—P2—Fe1	109.34 (6)	C14—C15—H15A	110.0
C11—P2—Fe1	118.47 (7)	O4—C15—H15B	110.0
C20—P3—C3	102.18 (10)	C14—C15—H15B	110.0
C20—P3—C17	104.79 (10)	H15A—C15—H15B	108.4
C3—P3—C17	97.55 (10)	O4—C16—H16A	109.5
C20—P3—Fe1	119.80 (7)	O4—C16—H16B	109.5
C3—P3—Fe1	106.55 (7)	H16A—C16—H16B	109.5
C17—P3—Fe1	121.90 (7)	O4—C16—H16C	109.5
C26—P4—C4	102.21 (9)	H16A—C16—H16C	109.5
C26—P4—C23	102.07 (10)	H16B—C16—H16C	109.5
C4—P4—C23	104.67 (9)	C18—C17—P3	119.71 (15)
C26—P4—Fe1	120.08 (7)	C18—C17—H17A	107.4
C4—P4—Fe1	108.37 (6)	P3—C17—H17A	107.4
C23—P4—Fe1	117.48 (7)	C18—C17—H17B	107.4
C7—O1—C6	114.15 (19)	P3—C17—H17B	107.4
C10—O2—C9	112.74 (18)	H17A—C17—H17B	106.9
C13—O3—C12	112.2 (2)	O5—C18—C17	110.17 (18)
C15—O4—C16	112.6 (2)	O5—C18—H18A	109.6
C18—O5—C19	111.58 (18)	C17—C18—H18A	109.6
C22—O6—C21	111.39 (17)	O5—C18—H18B	109.6
C25—O7—C24	112.52 (17)	C17—C18—H18B	109.6
C28—O8—C27	111.06 (17)	H18A—C18—H18B	108.1
C2—C1—P1	106.94 (14)	O5—C19—H19A	109.5
C2—C1—H1A	110.3	O5—C19—H19B	109.5
P1—C1—H1A	110.3	H19A—C19—H19B	109.5
C2—C1—H1B	110.3	O5—C19—H19C	109.5
P1—C1—H1B	110.3	H19A—C19—H19C	109.5
H1A—C1—H1B	108.6	H19B—C19—H19C	109.5
C1—C2—P2	108.53 (13)	C21—C20—P3	116.59 (14)
C1—C2—H2A	110.0	C21—C20—H20A	108.1
P2—C2—H2A	110.0	P3—C20—H20A	108.1
C1—C2—H2B	110.0	C21—C20—H20B	108.1
P2—C2—H2B	110.0	P3—C20—H20B	108.1
H2A—C2—H2B	108.4	H20A—C20—H20B	107.3
C4—C3—P3	108.03 (14)	O6—C21—C20	107.60 (17)
C4—C3—H3A	110.1	O6—C21—H21A	110.2
P3—C3—H3A	110.1	C20—C21—H21A	110.2
C4—C3—H3B	110.1	O6—C21—H21B	110.2
P3—C3—H3B	110.1	C20—C21—H21B	110.2
H3A—C3—H3B	108.4	H21A—C21—H21B	108.5
C3—C4—P4	108.11 (13)	O6—C22—H22A	109.5
C3—C4—H4A	110.1	O6—C22—H22B	109.5
P4—C4—H4A	110.1	H22A—C22—H22B	109.5
C3—C4—H4B	110.1	O6—C22—H22C	109.5
P4—C4—H4B	110.1	H22A—C22—H22C	109.5
H4A—C4—H4B	108.4	H22B—C22—H22C	109.5
C6—C5—P1	117.48 (14)	C24—C23—P4	117.85 (15)
C6—C5—H5A	107.9	C24—C23—H23A	107.8
P1—C5—H5A	107.9	P4—C23—H23A	107.8
C6—C5—H5B	107.9	C24—C23—H23B	107.8
P1—C5—H5B	107.9	P4—C23—H23B	107.8
H5A—C5—H5B	107.2	H23A—C23—H23B	107.2
O1—C6—C5	107.35 (16)	O7—C24—C23	111.16 (18)
O1—C6—H6A	110.2	O7—C24—H24A	109.4
C5—C6—H6A	110.2	C23—C24—H24A	109.4
O1—C6—H6B	110.2	O7—C24—H24B	109.4
C5—C6—H6B	110.2	C23—C24—H24B	109.4
H6A—C6—H6B	108.5	H24A—C24—H24B	108.0
O1—C7—H7A	109.5	O7—C25—H25A	109.5
O1—C7—H7B	109.5	O7—C25—H25B	109.5
H7A—C7—H7B	109.5	H25A—C25—H25B	109.5
O1—C7—H7C	109.5	O7—C25—H25C	109.5
H7A—C7—H7C	109.5	H25A—C25—H25C	109.5
H7B—C7—H7C	109.5	H25B—C25—H25C	109.5
C9—C8—P1	119.28 (15)	C27—C26—P4	115.60 (14)
C9—C8—H8A	107.5	C27—C26—H26A	108.4
P1—C8—H8A	107.5	P4—C26—H26A	108.4
C9—C8—H8B	107.5	C27—C26—H26B	108.4
P1—C8—H8B	107.5	P4—C26—H26B	108.4
H8A—C8—H8B	107.0	H26A—C26—H26B	107.4
O2—C9—C8	105.55 (17)	O8—C27—C26	108.24 (16)
O2—C9—H9A	110.6	O8—C27—H27A	110.1
C8—C9—H9A	110.6	C26—C27—H27A	110.1
O2—C9—H9B	110.6	O8—C27—H27B	110.1
C8—C9—H9B	110.6	C26—C27—H27B	110.1
H9A—C9—H9B	108.8	H27A—C27—H27B	108.4
O2—C10—H10A	109.5	O8—C28—H28A	109.5
O2—C10—H10B	109.5	O8—C28—H28B	109.5
H10A—C10—H10B	109.5	H28A—C28—H28B	109.5
O2—C10—H10C	109.5	O8—C28—H28C	109.5
H10A—C10—H10C	109.5	H28A—C28—H28C	109.5
H10B—C10—H10C	109.5	H28B—C28—H28C	109.5
C12—C11—P2	119.17 (15)		
			
P2—Fe1—P1—C5	140.63 (8)	P1—C1—C2—P2	50.69 (16)
P4—Fe1—P1—C5	−38.31 (8)	C14—P2—C2—C1	−157.94 (14)
Cl2—Fe1—P1—C5	−127.90 (8)	C11—P2—C2—C1	95.55 (15)
Cl1—Fe1—P1—C5	51.32 (8)	Fe1—P2—C2—C1	−32.36 (15)
P2—Fe1—P1—C8	−88.10 (8)	C20—P3—C3—C4	−172.46 (14)
P4—Fe1—P1—C8	92.96 (8)	C17—P3—C3—C4	80.57 (15)
Cl2—Fe1—P1—C8	3.37 (8)	Fe1—P3—C3—C4	−45.98 (15)
Cl1—Fe1—P1—C8	−177.41 (8)	P3—C3—C4—P4	52.38 (16)
P2—Fe1—P1—C1	22.58 (7)	C26—P4—C4—C3	−164.32 (14)
P4—Fe1—P1—C1	−156.36 (7)	C23—P4—C4—C3	89.55 (15)
Cl2—Fe1—P1—C1	114.05 (7)	Fe1—P4—C4—C3	−36.56 (15)
Cl1—Fe1—P1—C1	−66.73 (7)	C8—P1—C5—C6	59.36 (18)
P1—Fe1—P2—C14	119.39 (9)	C1—P1—C5—C6	−42.70 (18)
P3—Fe1—P2—C14	−64.89 (9)	Fe1—P1—C5—C6	−162.48 (13)
Cl2—Fe1—P2—C14	26.70 (9)	C7—O1—C6—C5	−179.5 (2)
Cl1—Fe1—P2—C14	−152.94 (9)	P1—C5—C6—O1	−177.78 (14)
P1—Fe1—P2—C2	2.12 (7)	C5—P1—C8—C9	66.56 (18)
P3—Fe1—P2—C2	177.84 (7)	C1—P1—C8—C9	172.87 (17)
Cl2—Fe1—P2—C2	−90.57 (7)	Fe1—P1—C8—C9	−71.69 (18)
Cl1—Fe1—P2—C2	89.79 (7)	C10—O2—C9—C8	−174.59 (18)
P1—Fe1—P2—C11	−117.64 (9)	P1—C8—C9—O2	−177.44 (14)
P3—Fe1—P2—C11	58.08 (9)	C14—P2—C11—C12	−94.62 (19)
Cl2—Fe1—P2—C11	149.67 (9)	C2—P2—C11—C12	13.0 (2)
Cl1—Fe1—P2—C11	−29.97 (9)	Fe1—P2—C11—C12	135.13 (16)
P2—Fe1—P3—C20	−45.62 (8)	C13—O3—C12—C11	−179.2 (2)
P4—Fe1—P3—C20	133.28 (8)	P2—C11—C12—O3	−74.6 (2)
Cl2—Fe1—P3—C20	−137.51 (8)	C2—P2—C14—C15	−55.2 (2)
Cl1—Fe1—P3—C20	43.27 (8)	C11—P2—C14—C15	53.4 (2)
P2—Fe1—P3—C3	−160.70 (7)	Fe1—P2—C14—C15	−175.56 (17)
P4—Fe1—P3—C3	18.21 (7)	C16—O4—C15—C14	154.0 (3)
Cl2—Fe1—P3—C3	107.42 (7)	P2—C14—C15—O4	−174.0 (2)
Cl1—Fe1—P3—C3	−71.80 (7)	C20—P3—C17—C18	71.91 (18)
P2—Fe1—P3—C17	89.04 (8)	C3—P3—C17—C18	176.69 (17)
P4—Fe1—P3—C17	−92.06 (8)	Fe1—P3—C17—C18	−68.42 (18)
Cl2—Fe1—P3—C17	−2.85 (8)	C19—O5—C18—C17	−171.85 (19)
Cl1—Fe1—P3—C17	177.93 (8)	P3—C17—C18—O5	−62.4 (2)
P1—Fe1—P4—C26	−60.68 (8)	C3—P3—C20—C21	−40.69 (19)
P3—Fe1—P4—C26	123.61 (8)	C17—P3—C20—C21	60.61 (18)
Cl2—Fe1—P4—C26	32.12 (8)	Fe1—P3—C20—C21	−158.04 (14)
Cl1—Fe1—P4—C26	−148.24 (8)	C22—O6—C21—C20	−172.0 (2)
P1—Fe1—P4—C4	−177.42 (7)	P3—C20—C21—O6	178.90 (15)
P3—Fe1—P4—C4	6.87 (7)	C26—P4—C23—C24	−91.54 (18)
Cl2—Fe1—P4—C4	−84.62 (7)	C4—P4—C23—C24	14.69 (19)
Cl1—Fe1—P4—C4	95.02 (7)	Fe1—P4—C23—C24	134.91 (14)
P1—Fe1—P4—C23	64.33 (8)	C25—O7—C24—C23	84.0 (2)
P3—Fe1—P4—C23	−111.38 (8)	P4—C23—C24—O7	179.90 (14)
Cl2—Fe1—P4—C23	157.13 (8)	C4—P4—C26—C27	−56.39 (18)
Cl1—Fe1—P4—C23	−23.24 (8)	C23—P4—C26—C27	51.74 (18)
C5—P1—C1—C2	−177.30 (13)	Fe1—P4—C26—C27	−176.26 (13)
C8—P1—C1—C2	76.56 (15)	C28—O8—C27—C26	171.15 (19)
Fe1—P1—C1—C2	−48.80 (14)	P4—C26—C27—O8	176.70 (14)
